# Self versus Family Ratings of the Frontal Systems Behaviour Scale and Measured Executive Functions: Adult Outcomes following Childhood Traumatic Brain Injury

**DOI:** 10.1371/journal.pone.0076916

**Published:** 2013-10-08

**Authors:** Robert D. Barrett, Tracey L. McLellan, Audrey McKinlay

**Affiliations:** 1 Department of Psychology, University of Canterbury, Christchurch, New Zealand; 2 School of Psychology and Psychiatry, Monash University, Melbourne, Victoria, Australia; University Children's Hospital Tuebingen, Germany

## Abstract

Traumatic brain injury (TBI) frequently occurs during childhood and adolescence with long-term neuropsychological and behavioral effects. Greater personal awareness of injury is associated with better outcomes. However, personal awareness is often assessed using ratings obtained from family members or significant others. Surprisingly, the accuracy of family-ratings compared with self-ratings has not been well studied in the TBI population. Thus, the purpose of this study was to examine self versus family-ratings of frontal dysfunction and secondly, the association between self/family reported frontal dysfunction and measured executive function outcomes. A total of 60 participants, approximately 10 years post-TBI, comprised 3 groups including; moderate/severe TBI (N=26; mean age 22.9, SD=3.0), mild TBI (N=20; mean age, 21.7, SD=2.7), and control (N=14: mean age, 21.6, SD=3.7). Neuropsychological testing was used to obtain domain scores for executive function and working memory/attention for each participant, and nominated family members and participants with TBI were asked to complete the Frontal Systems Behaviour Scale (FrSBe), consisting of three sub-scales; apathy, disinhibition, and executive dysfunction. Using the FrSBe there was no significant difference between the groups in executive function score, but the moderate/severe and mild groups had significantly lower working memory/attention scores compared with the control group (*p*<0.05). Repeated measures analysis of variance showed higher self-ratings on all sub-scales compared with family in each group (*p*<0.05). Scores on executive function and working memory/attention domains correlated with self, but not family reported executive dysfunction. Self-rated executive dysfunction explained 36% of the variance in executive function (*p*<0.001). While agreement between self-rated and family-rated total FrSBe scores was significant in all groups (*p*<0.001), our results showed that self-ratings were of higher predictive utility for executive functioning compared with family ratings. Further, at 10 years post-TBI, patients show greater awareness of deficits compared with family who rate consistently closer to the normal functioning range.

## Introduction

Traumatic brain injury (TBI) is a common childhood and young adult injury sustained in approximately 18% of young people prior to 14 years of age [1]. TBI is also a principal cause of disability in young people, and is associated with significant long-term effects on neuropsychological, behavioural, social, and employment outcomes [2-5]. Greater awareness of injury in individuals with TBI is associated with more positive employment, social, and emotional well-being outcomes, and better treatment outcomes in general [6-9]. Assessing the degree of awareness of deficits in individuals with TBI is important as treatment and intervention decisions can often be based on or influenced by ratings gathered from significant others or family members [10-12]. In cases where data from patients is unable to be obtained, ratings from family members may have even greater influence on treatment and assessment of injury, particularly on psychosocial problems [9,13,14].

Previous studies have shown conflicting results regarding the agreement between self and other ratings [15]. Some studies have found discord between patient’s and other’s ratings. In some studies other’s ratings of problems are higher than patient ratings [10,16-19]. Conversely, some studies have shown high agreement between patient and others ratings [20-22]. Specifically, physical, motor, and somatic measures are frequently associated with good overall agreement between patient and other ratings [6,22], whereas communication, depression, aggression, memory and attention, and quality of life often show discrepancy [10,17,20]. Patients usually rate themselves as less impaired compared with others on cognitive and emotional measures, and with a lower quality of life compared with others. However, some studies have shown high agreement in psychosocial outcome and cognitive, behavioural, emotional, and memory functions [10,22]. Moreover, agreement has been shown to be related to item specificity and content (higher specificity leads to higher agreement), the nature of the outcome measure (more readily observable leads to higher agreement), the number of items (larger number of items leads to higher agreement) [19,23,24], and injury severity depending on the attribute measured [12,20]. Thus, solely relying on patient or significant other ratings as indicators of patient status may lead to inaccuracies.

Interestingly, executive function deficits are often the subject of higher disagreement between patients and family ratings, with patients rating themselves as less impaired compared with family ratings [19,22]. As stated above, a lack of awareness regarding deficits is associated with poor long-term outcomes. Thus, it is essential to evaluate the predictive utility of self versus family ratings in relation to executive and other cognitive outcomes, as these ratings are important in determining appropriate treatment and intervention in individuals with TBI. In addition, there is sparse data from long-term longitudinal studies examining agreement between self and family ratings 10 years post-injury [12], and previous longitudinal studies have not specifically examined frontal system dysfunction agreement in relation to frontal ability outcomes measures, such as working memory, attention, and executive function. Executive dysfunction is often found in individuals, and especially children and adolescents, with TBI as, neuroanatomically, the areas involved in executive functions such as the prefrontal cortex are surrounded by bony ridges of the skull and are vulnerable to impacts associated with TBI [18].

Thus, the aim of the current study was to examine the self versus family ratings of frontal behaviour and whether either or both of these ratings were associated with executive deficits, as measured by formal testing, in individuals with TBI at on average 10 years post-injury. We also wanted to examine the predictive utility of self and family ratings in relation to the executive outcome measures. Specifically, we administered the Frontal Systems Behaviour Scale (FrSBe), which has been demonstrated to be a valid measure of behaviour disturbances associated with damage to frontal-subcortical brain networks [25], to both self and family to assess awareness of deficits. Additionally, we summarized executive function and working memory/attention scores from standardized tests into domain scores to serve as outcome measures of frontal ability.

## Methods

Approval for this study was granted by the Upper South New Zealand Regional Ethics Committee, and written informed consent was obtained from individuals who had experienced a TBI and from an orthopedic comparison group. The data reported here are part of a larger study examining the adult outcomes of childhood TBI.

### Participants

Participants were recruited via an audit of the neurosurgeon’s historical files, an audit of Hospital Emergency Department and Admission Records, and by placing notices in the community. General inclusion criteria for the study were that each participant had experienced an injury event as a child aged 0-17 years, were now 18 years or older, and more than 5 years post injury. Inclusion criteria for the moderate/severe TBI group included the following: i) clinical diagnosis of moderate or severe TBI; ii) skull fracture, evidence of lesion on tomography; iii) cerebral haemorrhage or Post-Traumatic Amnesia (PTA) > 24 hrs. Participants were included in the mild TBI group if they met the following criteria: i) a clinical diagnosis of mTBI; ii) loss of consciousness (LOC) < 20 min; iii) Post-Traumatic Amnesia (PTA) < 1 h; iv) no evidence of skull fracture or lesion on tomography. Participants in the orthopaedic injury control group were included if they had fractured an arm or leg in childhood and were excluded if they had ever experienced a TBI event. Recruitment yielded three groups of participants: 62 individuals with moderate/severe childhood TBI (21 female) aged 18-30 years (*M* = 23.29, *SD* = 3.55); 62 individuals with mild childhood TBI (29 female) were aged 18-30 years (*M* = 22.31, *SD* = 2.78), and the 43 orthopaedic controls (24 female) were aged 18-27 years (*M* = 21.81, *SD* = 3.36). All participants were fluent in English. Each participant was asked to attend a 3 hour test session conducted at the University of Canterbury. At the conclusion of the testing session, each participant was asked to nominate a family member, where possible, to complete the questionnaires described below. Family members (Mod/severe TBI=54; Mild TBI=51; Control=36) were sent the questionnaires to complete and return (Mod/severe=26/54, 48.1%; Mild TBI=20/51, 39.2%; Control=14/43, 32.6%). Analyses presented here were conducted on this sub-sample of participants.

### Measures and procedure

Demographic characteristics including sex and age were collected using a structured interview. Information regarding injury severity was collected using a file review. Pre-morbid IQ (PVIQ) was estimated using the National Adult Reading Test (NART) [26-30]. An executive function domain score was estimated from the mean standardized scores of the following tests; the verbal fluency subtest of Delis Kaplan Executive Functioning System [31] which requires each participant to generate as many different words as they can in 60-seconds that begin with the letters F,A and S; the Colour-Word interference – inhibition subtest of the D-KEFS in which the participant is presented with a page containing the words “red,” “green,” and “blue” printed incongruently in red, green, or blue ink, and asked to say the colour of the ink in which each word is printed as quickly as possible without making mistakes; The Matrix Reasoning subtest of the Wechsler Abbreviated Scale of Intelligence [32], where each participant selects among five alternatives the correct item needed to complete a presented sequence of geometric figures and patterns; The copy phase of the Rey-Osterreith Complex Figure Test [33] was used to determine an organizational planning score based on Organizational Scoring System for Amnesiacs [34] (Kixmiller et al. 2000), which measures planning by scoring the way in which the replication was ordered and organized; and a Tower of London task presented and completed on a touch screen computer provided a measure of executive planning by measuring ability to transform the start state into the goal state by moving coloured balls across three graduated rods. A working memory/attention score was also estimated using the standardized scores of the Adaptive Digit Ordering Task [35] (DOT-A: Werheid et al., 2002), which provides a measure of verbal working memory and requires the participant to recall an increasing sequence of numbers in ascending order; and the Daneman and Carpenter Reading Span test [36] (DCRS: Daneman & Carpenter, 1980) where each participant is required to read aloud sentences consisting of eight to thirteen words, judge whether the sentence makes sense or not, and recall the last word in each of the sentences.

The FrSBe [25] was used to quantify behaviours associated with frontal lobe brain damage. It has demonstrated validity in the assessment of behavioural dysfunction and disturbances associated with frontal-subcortical circuitry damage and TBI. The total score consists of three sub-scales: apathy, disinhibition, and executive Dysfunction. The self-rated version consists of 46 items rated on a 5-point Likert scale that measure behaviour before illness or injury and at the present time. All scores were converted to T-scores corrected for age, education, and gender according to the FrSBe administration manual [25], T-scores < 60 are considered normal; scores of 60-64 are considered of borderline significance, and scores of 65 or above are considered clinically significant. Clinically significant scores on the apathy subscale suggest problems with initiation, psychomotor retardation, spontaneity, drive, persistence, loss of energy and interest, lack of concern about self-care, and/or blunted affective expression. Clinically significant scores on the disinhibition subscale suggest difficulties with inhibitory control, impulsivity, hyperactivity, social inappropriateness or lack of conformity to social convention, excessive emotional expression, emotional lability, explosiveness, and/or irritability. Clinically significant scores on the executive dysfunction subscale denote self-reported problems with sustained attention, working memory, organization, planning, future orientation, sequencing, problem solving, insight, mental flexibility, self-monitoring of on-going behaviour, and/or ability to benefit from feedback or modify behaviour following errors [25]. The current study measured the behaviours at the present time only as participants were injured during childhood and adolescence.

### Statistical analysis

Data are presented as mean ± SD. Differences between groups in demographic characteristics and behavioural measures were assessed using analysis of variance (ANOVA). To control for the younger age at injury of the mild TBI group, age at injury as used as a covariate in all relevant analyses. Repeated measures ANOVA was used to explore differences between self and family scores between the groups. Bonferroni post-hoc tests were used to control for multiple comparisons. Pearson correlations were completed to determine the relationships between the self and family rated FrSBe scores, PVIQ, and the executive function and working memory/attention domain scores, with logistic regressions subsequently employed to assess the predictive utility of self or family FrSBe ratings on the domain scores. The agreement between the self and family FrSBe total score was measured using the Gower coefficient of agreement [37]. Briefly, the Gower index expresses the average absolute discrepancy between pairs of observations (scaled relative to the maximum possible discrepancy), which is then re-expressed as a measure of agreement by subtracting this discrepancy value from 1. It varies between 0 and +1, where +1 indicates identity between the two sets of observations. Computation of the Gower index was implemented using the 'Gower' computer program version 1.1 (www.pbarrett.net/software.html). The Gower coefficient of agreement has been used and validated in various disciplines including ecology, veterinary science, and cognitive science [38-40]. Because there is no obvious hypothetical sampling distribution for Gower coefficients, a bootstrap procedure was employed to compute credibility intervals (the interval within which we might expect to observe 95% of all coefficients computed using the same sample size, number-type (integers), and same minimum and maximum possible data range as that for the observed coefficient). A total of 10,000 resamples, of the same sample sizes used in the present study, from a uniform random number distribution were undertaken, from which the empirical sampling distribution of coefficients was created, and against which the observed coefficient could be assigned a probability of occurrence (the significance test) and an appropriate credibility interval constructed. Full details of the exact procedure are contained in the Bootstrap software used to perform the procedure (Bootstrap Version 1.0, http://www.pbarrett.net/Bootstrap/Bootstrap.html). All statistical calculations, except those pertaining to the Gower calculations, were made using SPSS 18 (IBM Corp., Somers, NY, USA).

## Results

Patient demographic characteristics and self and family ratings as a function of injury status are shown in Table 1. There were no significant group differences for age or PVIQ (Table 1). There was a significant group difference for Sex and Age at injury, with fewer males in the control group compared with the moderate/severe group, and a significantly younger age at injury in the mild compared with the moderate/severe group.

**Table 1:** Demographic characteristics, FrSBe ratings, and cognitive domain scores as a function of injury status.

	Mod/Severe TBI (n=26)	Mild TBI (n=20)	Orthopaedic Control (n=14)
	*Mean (SD)*	*Mean (SD)*	*Mean (SD)*
No. Males (%)	18 (69)^a^	10 (50)	4 (40)
Age	22.9 (3.0)	21.7 (2.7)	21.6 (3.7)
Age at injury	11.1 (4.95)^b^	7.0 (4.6)	10.1 (3.0)
Pre-morbid IQ	98.7 (11.6)	101.4 (9.4)	105.2 (6.7)
Self-rated FrSBe			
Apathy	58.0 (12.0)	61.9 (15.3)	57.7 (10.4)
Disinhibition	56.4 (10.6)	65.3 (14.2)	53.6 (5.5)
Executive Dysfunction	64.6 (15.2)	65.8 (14.2)	54.9 (10.7)
Total	62.6 (13.1)	67.2 (15.9)	55.2 (8.3)
**Family-rated FrSBe**			
Apathy	53.6 (17.3)	45.6 (13.0)	50.0 (14.1)
Disinhibition	47.7 (16.0)	44.0 (14.2)	38.6 (10.6)
Executive Dysfunction	51.8 (12.5)	47.5 (12.2)	42.2 (8.4)
Total	51.4 (14.6)	45.2 (14.7)	42.0 (12.2)
Executive Function domain score	-0.01 (0.7)	0.02 (0.36)	0.23 (0.6)
Working memory/ attention domain score	-0.3 (0.79)	-0.08 (0.73)	0.64 (0.65)^c^

TBI = Traumatic Brain Injury, FrSBe = Frontal Systems Behavior Scale. ^a^ Moderate/severe group significantly different to Control group, ^b^ Moderate/severe group significantly different to Mild group, ^c^ Control group significantly different to Moderate/severe group and Mild group. All differences were significant at *p* < 0.05.

### Domain scores

Executive function and Working memory/attention domain scores, both measures of frontal ability, were compared among the groups. There was no significant difference in Executive function domain score, but Working memory/attention was significantly lower in the moderate/severe and mild groups compared with the control group (Table 1).

### Self and family FrSBe behavioural ratings

The moderate/severe group reported clinically significant levels of Executive dysfunction, as did the mild group who also reported clinically significant problems with Disinhibition and borderline impairment with Apathy. The total scores for both groups revealed borderline impairment and clinical dysfunction. The control group reported neither clinically significant nor borderline impairment across the three sub-scales (see Table 1).

A 2 Rating (self, family) × 3 Syndrome (apathy, disinhibition, executive dysfunction) repeated measures ANOVA was conducted to explore differences between the groups. There was a main effect of Syndrome *F*(2, 106) = 5.94, *p* = 0.01, and a main effect of Rating *F*(1, 53) = 56.41, *p* = 0.001, which was qualified by a significant interaction *F*(2, 106) = 7.17, *p* = 0.01. Post hoc testing (Bonferroni, *p* < .05) showed higher self-ratings (M_apathy_ = 59.2, SD = 12.8; M_disinhibition_ = 58.7, SD = 11.9; M_executive dysfunction_ = 62.7, SD = 14.5) than family ratings (M_apathy_ = 50.0, SD = 15.3; M_disinhibition_ = 44.3, SD = 14.5; M_executive dysfunction_ = 50.0, SD = 12.0) for all 3 syndromes. Furthermore, self-ratings revealed higher Executive dysfunction than problems with Apathy or Disinhibition which did not differ from each other, while family ratings revealed more Apathy as well as Executive dysfunction than problems with Disinhibition.

There was no main effect of Group, although there was a significant interaction with Syndrome *F*(4, 106) = 3.35, *p* < 0.05 which post hoc testing (Bonferroni, *p* < .05) showed due to significantly higher levels of Executive dysfunction (*M* = 58.1, *SD* = 13.4) than Disinhibition (*M* = 51.6, *SD* = 11.2) in the Moderate/Severe Group and significantly higher levels of Apathy (*M* = 53.8, *SD*= 12.4) than Disinhibition (*M* = 46.1, *SD* = 8.5) in the control group. No difference between the syndromes was found in the Mild group.

Group also significantly interacted with Rating *F*(2, 53) = 3.24, *p* < 0.05. Significantly higher self-ratings compared to family ratings were found for each Group: Moderate/severe (*M* = 59.1, *SD* = 13.0 vs. *M* = 50.9, *SD* = 14.4), Mild (*M* = 64.3, *SD* = 13.9 vs. *M* = 46.3, *SD* = 14.7), and Control (*M* = 55.4, *SD* = 8.3 vs. *M* = 43.6, *SD* = 12.2).

### Correlations among self-reported and family-reported FrSBe scores and domain scores

Person correlation was used to detect relationships among self and family rating FrSBe scores and cognitive domain scores, i.e. among reported and measured estimates of frontal functioning. As can be seen in Table 2 both Executive function and Working memory/attention were significantly correlated with self-reported Executive Dysfunction, but not family-reported Executive Dysfunction. The family scores did not correlate with either of the measured domain scores. Self-reported Disinhibition also correlated with Working memory/attention. Each of the self-reported scores correlated to the corresponding family-reported score.

**Table 2:** Correlations between domain scores, self-rated, and family-rated frontal syndromes.

	1	2	3	4	5	6	7	8
1) Executive function	1							
2) Working memory/attention	.55**	1						
3) Self-reported apathy	-.25	-.19	1					
4) Self-reported disinhibition	-.26	-.28*	.60**	1				
5) Self-reported exec dysfunction	-.50**	-.39**	.57**	.71**	1			
6) Family-reported apathy	-.14	-.05	.26*	.15	.19	1		
7) Family-reported disinhibition	-.05	-.13	.22	.46**	.24	.65**	1	
8) Family-reported exec dysfunction	-.20	-.16	.49**	.44**	.48**	.74**	.68**	1

* *p* < 0.05, **p < 0.01 (2-tailed)

### Domain score predictive utility of self versus family FrSBe score

Regression analysis was performed to examine the predictive utility of self-rated FrSBe scores in explaining the variance in measured Executive function. The total variance explained was 36%, *F*(2, 55) = 15.39, *p* < .001. Of this, self-reported Executive Dysfunction explained a unique 11% *F*(1, 55) = 9.50, *p* < .01, having accounted for PVIQ, and was a significant predictor of measured Executive function (beta = .360, *p* < .001). Disinhibition was added to the model for Working memory/attention. The total variance explained was 41%, *F*(3, 54) = 12.29, *p* < .001. Executive Dysfunction and Disinhibition, however, did not significantly account for variance in Working memory/attention scores over and above that explained by PVIQ.

### Agreement between self and family total FrSBe score

The Gower coefficient of agreement was used to assess how discrepant or identical self versus family ratings of the FrSBe total score were. The details and procedure for testing the significance of the coefficients are detailed in the methods section. All three groups had a significantly high coefficient of agreement (Control group, 0.85, *p* = 0.001, 95% CI 0.54-0.79; Mild group, 0.82, *p* = 0.001, 95% CI 0.56-0.77; Moderate/severe group, 0.85, *p* < 0.000, 95% CI 0.57-0.76), indicating that self and family ratings agreed to within a significantly high percentage of each other’s values in each group (i.e. 85%, 82% and 85% for the Mild, Moderate/severe, and control groups, respectively). Interestingly, inspection of discrepancy histograms for the mild group (Self-Family) showed that in all cases the family FrSBe total score was lower compared with the self-rating score, indicating that all mild group participants rated themselves as more dysfunctional compared with family ratings. In contrast, there were two (14%) negative discrepancies in the control group, indicating family rating scores exceeded self-rating scores, and in the moderate/severe group six (23%) cases had a family score higher than the self-rating score.

## Discussion

Assessing the awareness of deficits in TBI is important, as those who are unaware of deficits are more likely to have problems finding employment, maintaining relationships, and have vocational and other social problems [6,41,42]. In the current study, young adults who experienced an injury during their childhood and their family member were administered the Frontal Systems Behaviour Rating Scale (FrSBe) to report behaviour on average 10 years post-injury. In addition, the executive function and working memory/attention skills of these young adults was also tested to measure current functioning.

As expected, no clinically significant difficulties with frontal skills were observed in the control group, either through ratings or testing. Interestingly, the mild group self-reported clinically significant disinhibition and executive Dysfunction, whereas the moderate/severe group only reported this level of problem with executive Dysfunction. Overall, we found individuals with a history of TBI had difficulty with specific frontal skills compared with controls. Specifically, long-term working memory/attention deficits were found. We also found that while individuals with a history of TBI tended to self-report clinically significant problems with frontal functioning, their family member did not. Indeed, it was self-ratings that were found to significantly correlate with measured frontal ability. These results suggest that those with a history of TBI demonstrate an awareness of difficulty that people close to them may not see. Although similar difficulties within the domain of executive functioning were not found, Pearson correlation and regression showed self-ratings were of higher predictive utility than family ratings in accounting for variance.

This is in line with previous research that found participants rated themselves as more deficient than family did [17,20]. Interestingly, Seel et al. [20] also reported that mild as well as moderate/severe injury patients rated problems occurring more frequently compared with their family members. Our results are also consistent with reports showing that with increasing time post-injury, self-awareness becomes more accurate [12,13,16,43]. Moreover, most previous reports studied participants two years or less post-injury, whereas the current study, like [13] assessed participants at approximately 10 years post-injury. Thus, it is unsurprising that participants in the current study showed a greater awareness of injury compared with their family.

The agreement data confirmed that although the level of agreement was high, family report less difficulty than self and less difficulty than actually present. Thus, clinicians and researchers should be mindful of using only family ratings when drawing conclusions about cognitive and neurobehavioral deficits, and instead seek perspective from multiple sources [13]. Thus, the results suggest that self-ratings of the FrSBe, approximately 10 years after injury, are of greater diagnostic utility compared with family ratings, but family do demonstrate some awareness, although report ratings closer to the normal functioning non-clinical range. This is an important finding as previous studies using the Neurobehavioral Functioning Inventory (NFI) have stressed that given its greater focus on physical impairments following TBI, the NFI may not be the most appropriate measure in long-term studies, and it does not assess residual problems with executive function [12]. In contrast, the FrSBe is designed to assess behavioural disturbances associated with damage to frontal and subcortical connections and TBI. Thus, the FrSBe is a more relevant measure in long-term studies, particularly with greater understanding of the role of self and family reports.

Some limitations of the current study should be noted. Firstly, the sample size was small due to a low response rate from family members. A larger study whereby family information is collected on location would help reduce this potential bias. To check that our sample was representative of the full sample, domain scores from patients with responding family members were compared with a sample from non-responders and no significant differences were found. There was no opportunity in the current study to perform precise morphometric measurements and analyses of lesion location, volume, or network connectivity, or to investigate the relationship of these measures to domain score outcomes; such investigations must be the subject of further studies to extend the present results with neurobiological correlates. It is possible that the self-report nature of the FrSBe measure may have been biased by the participant’s reluctance to divulge negative information about themselves. However, it would be expected that this problem would be consistent across the groups. In contrast, the elevated scores in the mild TBI group may have been influenced by demand characteristics, as those in the mild TBI group potentially report symptoms and experiences to meet the expectation that their injury status/history reveals problematic behaviours. Lastly, caution should be used in interpreting outcomes given the younger age at injury of the mild TBI group. However, age at injury was used as a covariate in relevant analyses to control for any effects of the younger age at injury of the mild TBI group.

The findings from this study have implications for clinical practice and intervention. Clinicians should be mindful that self report and family report are both valid, but different sources of information and it should not be expected that they will be congruent. Information from both sources should routinely be taken. The findings of this study were undertaken 10 years post-injury, but represent a single time point. A longitudinal perspective on this topic with information being collected at multiple time points post-injury would provide further information regarding self-awareness and how this skill is best rehabilitated following TBI.

## Summary

In summary, this study has shown that while there was high agreement between individuals with TBI and their family members on a test of frontal systems related behavioural problems, family members consistently rated the individual with TBI as less impaired. Overall our results demonstrate that self-rated FrSBe scores have greater predictive utility for performance on executive function and working memory/attention. These results demonstrate that individuals 10 years post TBI have greater awareness of deficits compared with family members. In terms of clinical application, it should be noted that individuals with TBI may have more insight into their problems than family members.
